# *Staphylococcus aureus* endocarditis: Identifying prognostic factors using a method derived from morbidity and mortality conferences

**DOI:** 10.3389/fmed.2022.1053278

**Published:** 2022-12-06

**Authors:** Benjamin Lefèvre, Antoine Legoff, Mathilde Boutrou, François Goehringer, Willy Ngueyon-Sime, Catherine Chirouze, Matthieu Revest, Véronique Vernet Garnier, Xavier Duval, François Delahaye, Vincent Le Moing, Christine Selton-Suty, Laura Filippetti, Bruno Hoen, Nelly Agrinier

**Affiliations:** ^1^Université de Lorraine, CHRU-Nancy, Service des Maladies Infectieuses et Tropicales, Nancy, France; ^2^Université de Lorraine, APEMAC, Nancy, France; ^3^CH Andrée Rosemon, Unité de Maladies Infectieuses et Tropicales, Cayenne, France; ^4^CHRU-Nancy, Service des Maladies Infectieuses et Tropicales, Nancy, France; ^5^CHRU-Nancy, Institut National de la Sante et de la Recherche Medicale (INSERM), Université de Lorraine, CIC, Epidémiologie Clinique, Nancy, France; ^6^UMR 6249 CNRS-UFC Chrono-environnement, Service de Maladies Infectieuses, CHRU Besançon, Besançon, France; ^7^Infectious Diseases and Intensive Care Unit, Pontchaillou University Hospital, Rennes, France; ^8^CIC-Institut National de la Sante et de la Recherche Medicale (INSERM) 1414, Pontchaillou University Hospital, Rennes, France; ^9^University of Rennes, Institut National de la Sante et de la Recherche Medicale (INSERM), Bacterial Regulatory RNAs and Medicine, UMR 1230, Rennes, France; ^10^UFR Médecine, CHU Robert Debré, Reims, France; ^11^Institut National de la Sante et de la Recherche Medicale (INSERM) CIC 1425, Bichat–Claude Bernard Hospital, Assistance Publique-Hôpitaux de Paris, Paris, France; ^12^Institut National de la Sante et de la Recherche Medicale (INSERM), UMR-1137, IAME, Paris University, Paris, France; ^13^Université de Paris, IAME, Institut National de la Sante et de la Recherche Medicale (INSERM), Paris, France; ^14^Centre for Clinical Investigation, Assistance Publique-Hôpitaux de Paris, Bichat-Claude Bernard University Hospital, Paris, France; ^15^Louis Pradel Hospital, Department of Cardiology, Lyon, France; ^16^Montpellier University Hospital, Department of Infectious and Tropical Diseases, Montpellier, France; ^17^CHU Nancy-Brabois, Department of Cardiology, Nancy, France

**Keywords:** *Staphylococcus aureus*, infective endocarditis, prognostic factors, survival, morbidity and mortality conference method

## Abstract

**Objectives:**

Lethality of *Staphylococcus aureus* (Sa) infective endocarditis (IE) is high and might be due to yet unidentified prognostic factors. The aim of this study was to search for new potential prognostic factors and assess their prognostic value in SaIE.

**Materials and methods:**

We used a two-step exploratory approach. First, using a qualitative approach derived from mortality and morbidity conferences, we conducted a review of the medical records of 30 patients with SaIE (15 deceased and 15 survivors), randomly extracted from an IE cohort database (NCT03295045), to detect new factors of possible prognostic interest. Second, we collected quantitative data for these factors in the entire set of SaIE patients and used multivariate Cox models to estimate their prognostic value.

**Results:**

A total of 134 patients with modified Duke definite SaIE were included, 64 of whom died during follow-up. Of the 56 candidate prognostic factors identified at the first step, 3 had a significant prognostic value in multivariate analysis: the prior use of non-steroidal anti-inflammatory drugs [aHR 3.60, 95% CI (1.59–8.15), *p* = 0.002]; the non-performance of valve surgery when indicated [aHR 1.85, 95% CI (1.01–3.39), *p* = 0.046]; and the decrease of vegetation size on antibiotic treatment [aHR 0.34, 95% CI (0.12–0.97), *p* = 0.044].

**Conclusion:**

We identified three potential SaIE prognostic factors. These results, if externally validated, might eventually help improve the management of patients with SaIE.

## Introduction

Infective endocarditis (IE) is a rare but serious disease associated with high morbidity and mortality (1, 2). *Staphylococcus aureus* (Sa) became the most frequent pathogen responsible for IE during the past decades (3, 4). In comparison with other pathogens, lethality of SaIE is higher (4, 5). Its morbidity is also of concern, with 20–60% of embolic events (4, 5), 20–25% of sepsis (6, 7), and 26–38% of cardiac surgery during the initial management (4, 5).

To improve the outcomes of SaIE, identifying new prognostic factors may help tailor IE management to patients’ risk and improve patients’ management accordingly. Prognostic factors have already been identified in IE and SaIE. Among these, patients’ characteristics such as age (4, 8) and comorbidities (1, 8), have a major prognostic impact. Many SaIE clinical characteristics are associated with lethality, including prosthetic valve infection (8, 9), disease severity (septic shock and SOFA score) (9, 10), and heart, valve, or organ failures (1, 11). Higher mortality is also associated with left-sided IE (12), intracardiac abscess (1, 8), and larger vegetations (1, 13). Embolic events (2, 13), neurological complications (1, 10), and cardiac conduction abnormalities (14) are associated with poorer outcome. Methicillin-resistance of Sa (9) and persistent bacteremia are also associated with poorer outcome (4). Finally, deviations from optimal management such as inadequate antibiotic therapy (15) and delayed surgical treatment (8, 16) may also worsen the prognosis of SaIE. The plateauing high mortality observed in SaIE over the last decades (4, 5, 17, 18) suggests that specific prognostic factors may have not yet been identified. Moreover, most of the already identified prognostic factors are not modifiable. Searching for new prognostic factors might lead to identify modifiable factors and help design interventions that could improve SaIE outcome.

Prognostic factors are best identified using longitudinal studies and collecting candidate prognostic factors at baseline and outcomes during the follow-up, and then using Cox models to estimate the prognostic value of each factor. However, such methods require having candidate factors before initiating data collection. Once major factors, such as age and comorbidities, have been tested, identification of newer candidate factors becomes harder. Morbidity and mortality conferences (MMC) are designed to improve the quality of healthcare by addressing errors in patient’s management. In these conferences, healthcare professionals review and discuss, collegially, confidentially, and critically, the charts of patients who developed severe adverse events, to identify healthcare related factors that contributed to their occurrence (19). We thought that this approach could be helpful to identify candidate prognostic factors in SaIE. The aim of this study was to search for new potential prognostic factors and assess their prognostic value in SaIE.

## Materials and methods

### Study design

We used an exploratory two-step approach that included: (1) a qualitative approach derived from MMC to identify candidate prognostic factors in SaIE, and (2) a conventional quantitative approach to estimate the prognostic value of the factors identified at the end of step 1, using the source data that had been collected for the EI2008 study (NCT03295045) (3).

### Setting

The EI2008 study has been extensively described elsewhere (3). Briefly, EI2008 is a longitudinal cohort study conducted in France in 2008, which aimed to describe IE incidence and prognosis, and enrolled 602 patients aged ≥ 18 years with definite (*n* = 497) or possible (*n* = 105) IE according to modified Duke criteria. Data collection consisted of demographic characteristics, medical history, medications, IE mode of acquisition, and clinical, biological, and therapeutic characteristics (see Supplementary material). Patients were followed up for 1-year all-cause mortality (3). The identification of all Sa strains had been confirmed by the national reference center for staphylococci (Centre National de Référence des Staphylocoques, Institut des Agents Infectieux, Hospices Civils de Lyon, Lyon, France).

### Participants

For the first-step qualitative approach, among 134 patients with modified Duke definite SaIE included in EI2008, we used computer-generated random numbers to sample 15 patients with definite SaIE who had died and 15 patients with definite SaIE who had survived at the end of the 1-year follow-up.

**FIGURE 1:**
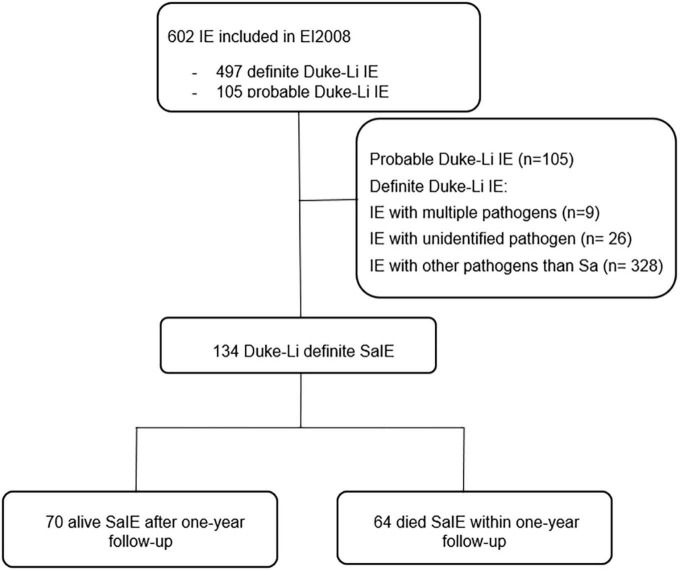
Flow chart of patients with *Staphylococcus aureus* infective endocarditis (SaIE) selected from EI2008. IE, infective endocarditis, Sa, *Staphylococcus aureus*.

For the quantitative approach, we selected from the EI2008 study database all the 134 patients with modified Duke definite SaIE, of whom 70 survived and 64 died during follow-up (Figure 1).

### Data collection

For the qualitative approach, we used a method inspired by MMC (19, 20) and conducted an in-depth review of the whole patients’ charts (MB, AL, and BL) to identify factors that were not already collected in the EI2008 case report form (CRF) but could be associated with the outcome (defined as “candidate prognostic factors”). We searched the charts of the 15 patients who died during follow-up for factors deemed unfavorable candidate prognostic factors, and the charts of the 15 patients who survived for factors deemed favorable candidate prognostic factors. Following the guidelines of the French National Authority for Health on MMC (20), each candidate prognostic factor was then collegially and critically reviewed (by MB, AL, BL, NA, and BH) to drop duplicates (i.e., variables that were already collected as part of EI2008 CRF) and to select those that were deemed relevant on a clinical and pathophysiological perspective. All the candidate prognostic factors that were retained at the end of the first step were collected from the medical charts of all the 134 patients included in the quantitative approach and were implemented into an enriched EI2008 database.

For the quantitative approach, we re-used the data that were prospectively collected as part of the EI2008 cohort study protocol and additional data (concerning the candidate prognostic factors identified by the qualitative approach) that were specifically collected from patients’ medical charts for the purpose of the present study.

### Statistical analyses

We first described patients’ characteristics and the distribution of candidate factors, using frequencies and percentages for categorical variables, or median and interquartile range for quantitative variables. Second, we used Cox models to identify potential prognostic factors among candidates using bivariate analyses. We retained candidate prognostic factors with a *p*-value < 0.2 as eligible for multivariate analyses. Third, for each eligible candidate prognostic factor, we conducted a multivariate Cox model, using the given factor as an independent variable and adjusting for confounding factors that were identified among baseline characteristics and candidate prognostic factors by their bivariate association (*p*-value < 0.2) with both survival and the candidate prognostic factor under scrutiny. *P*-values were two-sided, and statistical significance level was set at 0.05. All statistical analyses were performed using SAS 9.4 (SAS Institute, Inc., Cary, NC, USA) software.

### Ethics

This study complies with the principles outlined in the Declaration of Helsinki. EI2008 was approved by the French Commission Nationale Informatique et des Libertés (CNIL-DR-2010-219). Patients received complete information about the study, and their right to refuse to participate. EI2008 being an observational study, in accordance with the French law, patient written consent was waived (NCT03295045).

## Results

### *Staphylococcus aureus* infective endocarditis patients’ characteristics and follow-up

The characteristics of the 134 patients are described in Table 1. Their median age was 62.5 years (IQR 31 years) and there was a majority of men (*n* = 98, 73.1%). A heart disease at risk of IE was present in 54 (40.3%) patients. Most IE cases were community-acquired (*n* = 72, 53.7%). Most frequent complications included septic shock (*n* = 49, 36.6%), cerebral embolisms (*n* = 48, 35.8%), and heart failure (*n* = 13, 9.7%).

**TABLE 1:** Main characteristics of the 134 patients with *Staphylococcus aureus* infective endocarditis.

Patient’s characteristics		*N*	%	Median	Q1	Q3
**Sociodemographic**						
Age (years)		134	1	62.5	44.0	75.0
Women		36	26.9			
**Medical history**						
Obesity		21	15.7			
Heart disease without risk of IE		67	50.0			
Heart disease at risk of IE		54	40.3			
Native valve disease		38	28.4			
Intra-cardiac device		20	14.9			
Infective endocarditis		6	4.5			
Smoker		31	23.1			
Alcohol abuse		20	14.9			
Anticoagulant or antiplatelet therapy		56	41.8			
Charlson comorbidity index		134	1	1.0	0.0	3.0
**Clinical characteristics**						
Acquisition mode	Community-acquired	72	53.7			
	Healthcare-associated	39	29.1			
	Intravenous drug use	23	17.2			
Initial Glasgow score		112	83.6	15.0	14.0	15.0
Heart failure		13	9.7			
Cardiac conduction abnormalities		14	10.4			
Arterial aneurysm		4	3.0			
Vertebral osteomyelitis		13	9.7			
Arthritis		16	11.9			
Septic shock and sepsis		49	36.6			
Extra-cardiac device infection		12	9.0			
Acute kidney injury		21	15.7			
**Embolic complications**						
Cerebral		48	35.8			
Peripheral		61	41.5			
**Biological characteristics**						
White blood cells (G/L)		131	97.8	13.1	9.3	16.8
CRP (mg/L)		123	91.8	226.0	131.0	316.0
GFR (ml/min/1.73 m^2^)		129	96.3	57.6	34.1	80.8
Methicillin resistant Sa		17	12.7			
**Echocardiographic**						
Vegetation	Size > or = 10 mm	73	54.5			
	Size < 10 mm	23	17.2			
Location	Aortic	39	29.1			
	Mitral	52	38.8			
	Tricuspid	22	16.4			
	CIED	7	5.2			
Intra-cardiac abscess		21	15.7			
New native valve regurgitation/perforation		110	82.1			
Prosthetic endocarditis		17	12.7			
New prosthetic regurgitation/dehiscence		6	4.5			
**Therapeutics**						
Heart surgery and/or CIED removal		62	46.3			
Care or surgical complications		30	22.4			
Extra-cardiac surgery		8	6.0			

Frequencies and percentages used for categorical variables. Median and interquartile range used for quantitative variables. IE, infective endocarditis; Sa, *Staphylococcus aureus*; CRP, C-Reactive Protein; GFR, Glomerular Filtration Rate; CIED, cardiac implantable electronic device.

### Identification of candidate prognostic factors in *Staphylococcus aureus* infective endocarditis: Results of the qualitative approach

In the charts of the 15 surviving patients, we identified 22 candidate prognostic factors that could be associated with higher survival (Table 2). In the charts of the 15 deceased patients, we identified 34 candidate prognostic factors that could be associated with higher mortality (Table 3). All these candidate prognostic factors were then collected in the medical charts of all the 134 patients included in the quantitative approach (the frequency of each factor was presented in Tables 2, 3).

**TABLE 2:** Description of candidate prognostic factors that could be associated with survival in the 134 *Staphylococcus aureus* infective endocarditis patients.

	Definition	*N*	%
**Patient medical history**			
No antibiotic allergy	No history of allergy whatever the class of antibiotic	125	93.3
Previous Sa infection	Prior history of documented Sa infection	6	4.5
Cardiovascular treatment	Drug with cardiovascular effects (antiplatelet therapy, anticoagulant, diuretic, antiarrhythmic, RAAS inhibitors, statins, antihypertensive drug)	61	45.5
**Severity upon admission**			
Immunological sign of IE	Splenomegaly, glomerulonephritis, purpura, Osler nods	22	16.4
Initial neurological sign	Neurological signs of IE at the first medical examination	50	37.3
Sa susceptible strain	Sa strain susceptible to any antibiotic or resistant only to penicillin G	83	61.9
Penicillin G susceptible Sa strain	*In vitro* phenotypic Sa susceptibility against penicillin G	13	9.7
Low blood inflammation	First blood analysis: CRP < 150 mg/L + WBC < 15 G/L + neutrophils < 10 G/L	16	11.9
**Healthcare management**			
Ophtalmologist exam	Posterior segment eye examination was performed during hospitalization	5	3.7
Low initial inoculum	Only one positive blood culture among blood culture performed before antibiotic initiation	9	6.7
Early first echocardiography	Echocardiography ≤ 48 h of hospitalization or after first positive blood culture	90	67.2
Decreasing vegetation size on antibiotic treatment	Evidence of decreasing vegetation size on antibiotic treatment (at least one millimeter) when comparing diagnosis echocardiography and last in-hospital echocardiography (censored by surgery in operated patient)	23	17.2
Early body computed tomography	Computed tomography during the first week of hospitalization or after first positive blood culture	64	47.8
Prolonged antibiotic combined therapy	Concomitant administration of two different antibiotics ≥ 14 days	95	70.9
Extracardiac infectious surgery	Surgery of an extracardiac infected site to control inoculum	27	20.1
Steroid use	Use of steroid during the hospitalization	15	11.2
**Hospital organization**			
IE team	Management involving an IE team as described by ESC guidelines (15)	101	75.4
Early IE team	Management involving an IE team during the first week of hospitalization	53	39.6
Hospitalization report signed by a senior physician	Hospitalization report signed by a physician with a permanent position	97	72.4
Records completed by a physician	Hospitalization records completed by a physician with a permanent position	27	20.5
**Outcomes of hospitalization**			
CIED implantation	CIED implantation during hospitalization	12	9.0
Oral antibiotic switch	Oral antibiotic switch during hospitalization	37	27.6

Frequencies and percentages used for categorical variables. IE, infective endocarditis; Sa, *Staphylococcus aureus*; RAAS, renin angiotensin aldosterone system; CRP, C-Reactive Protein; WBC, white blood cells; ESC, European Society of Cardiology; CIED, cardiac implantable electronic device.

**TABLE 3:** Description of factors that could be associated with death in the 134 *Staphylococcus aureus* infective endocarditis patients.

	Definition	*N*	%
**Patient medical history**			
Prolonged bed rest	Uninterrupted bed rest ≥ 48 h	12	9.0
Patient dependent for ADL	Patient needing assistance for eating, bathing, getting dressed, toileting, mobility, and continence	10	7.5
Patient dependent for IADL	Patient needing assistance for cooking, cleaning, transportation, laundry, and managing finances	10	7.5
Skin pressure ulcer	Skin pressure ulcer before hospitalizations for SaIE	12	9.0
Penicillin allergy	Penicillin allergy known at hospital admission	6	4.5
COPD	Documented history of COPD	8	6.0
Prior use of NSAIDs	Use of NSAIDs during the week before the hospital admission	10	7.5
MRSa carriage	Documented history of carriage of MRSa at hospital admission	1	0.7
**Severity upon admission**			
Rifampicin resistance	Sa strain with *in vitro* phenotype of rifampicin resistance	2	1.5
Quinolone resistance	Sa strain with *in vitro* phenotype of quinolone resistance	10	7.5
Use of vasoactive drug	Use of adrenaline or noradrenaline during the hospitalization	37	27.6
Use of dobutamine	Use of dobutamine during the hospitalizations	16	11.9
**Healthcare management**			
No probabilistic antibiotic treatment (PAT)	No use of a PAT before Sa identification	50	37.3
No efficient PAT	No use of a PAT with an efficient (suboptimal efficacy) scope before Sa identification	62	46.3
No optimal PAT	No use of a PAT with an optimal (antibiotic with the higher proved efficacy) scope before Sa identification	119	88.8
Not recommended antibiotic treatment	Antibiotic therapy did not follow ESC guidelines (15) more than half the treatment duration	49	36.6
Gentamycin	Use of at least one dose of gentamycin during hospitalization	100	74.6
Vancomycin	Use of at least one dose of vancomycin during hospitalization	59	44.0
Oxacillin	Use of at least one dose of oxacillin during hospitalization	90	67.2
Rifampicin	Use of at least one dose of rifampicin during hospitalization	68	50.7
Quinolone	Use of at least one dose of quinolone during hospitalization	68	50.7
Persistent deep infected site	Deep infected site that persisted in the absence of surgical act	28	20.9
CIED Implantation during septic period	CIED is implanted before infection control proved by negative blood culture	10	7.5
Non-performance of valve surgery when indicated	No valve surgery was performed during hospitalization despite indication as described in ESC guidelines (15) (Death, patients’ refusal, and contraindications)	26	19.4
Time before surgery respected	Time before the valve surgery according to the level of emergency	43	32.1
Extracardiac surgery indication	Indication of an extracardiac surgery to control the Sa infection	28	20.9
Palliative care	Current Sa infection severity leading to a decision to limit active care	22	16.4
**Hospital organization**			
Unavailable extra-cardiac surgery	Extracardiac surgery was indicated but not offered in the concerned hospital	5	3.7
Unavailable cardiac surgery	Cardiac surgery was indicated but not offered in the concerned hospital	12	9.0
**Outcomes of hospitalization**			
Adverse event related to anticoagulant	A hemorrhagic adverse event due to anticoagulant use occurred	6	4.5
Surgical complication	An adverse event due to surgery act occurred whatever its form	10	7.5
Nosocomial infection	When a healthcare associated infection occurred during hospitalizations	25	18.7
Antibiotic allergy	An anaphylactic adverse event due to antibiotic occurred	9	6.7
Antibiotic toxicity	When an adverse event due to antibiotic occurred, whatever is form	23	17.2

Frequencies and percentages used for categorical variables. IE, infective endocarditis; Sa, *Staphylococcus aureus*; COPD, chronic obstructive pulmonary disease; NSAIDs, non-steroidal anti-inflammatory drugs; MRSa, methicillin resistant *Staphylococcus aureus*; PAT, probabilistic antibiotic treatment (antibiotic treatment when endocarditis is suspected and not proved by microbiologist analysis); ESC, European Society of Cardiology; CIED, cardiac implantable electronic device.

Candidate prognostic factors were sorted out into five categories: patient medical history (3 potentially associated with higher survival vs. 8 potentially associated with higher mortality), severity upon admission (5 vs. 4), healthcare management (8 vs. 15), hospital organization (4 vs. 2), and outcomes of hospitalization (2 vs. 5).

### Prognostic value of candidate prognostic factors in *Staphylococcus aureus* infective endocarditis: Results from the quantitative approach

Eligible factors associated with outcome are presented in Table 4. After adjustment for potential confounders, two eligible candidate prognostic factors remained significantly associated with higher mortality during the 1-year follow-up: the prior use of non-steroidal anti-inflammatory drugs (NSAIDs) [aHR 3.60, 95% confidence interval (95% CI) (1.59–8.15), *p* = 0.002] and the non-performance of valve surgery when indicated [aHR 1.85, 95% CI (1.01–3.39), *p* = 0.046]. One eligible candidate prognostic factor was associated with lower mortality: the decrease of vegetation size on antibiotic treatment [aHR 0.34 95% CI (0.12–0.97), *p* = 0.044].

**TABLE 4:** Prognostic value of factors that could be associated with *Staphylococcus aureus* infective endocarditis outcome.

	One-year follow up mortality				Bivariate analysis			Multivariate analysis			
			
		*n*	rate	95% CI	HR	95% CI	*P*-value	aHR	95% CI	*P*-value	Adjustment variables
**Factors that could be associated with survival**											
**Patient medical history**											
Cardiovascular treatment	Yes	47	0.66	0.55–0.76	3.06	1.75–5.34	<0.001				
	No	17	0.28	0.18–0.41	Ref						
**Severity upon admission**											
Immunological sign of IE	Yes	6	0.27	0.13–0.51	0.43	0.19–1.01	0.052				
	No	58	0.52	0.43–0.62	Ref						
Initial neurological sign	Yes	37	0.75	0.62–0.86	3.67	2.22–6.05	<0.001				
	No	27	0.32	0.24–0.44	Ref						
**Healthcare management**											
Low initial inoculum	Yes	2	0.22	0.06–0.64	0.35	0.09–1.44	0.147				
	No	62	0.50	0.42–0.59	Ref						
Early first echocardiography	Yes	46	0.51	0.42–0.62	1.58	0.91–2.72	0.102				
	No	18	0.42	0.29–0.58	Ref						
Decreasing vegetation size on antibiotic treatment	Yes	4	0.18	0.07–0.41	0.23	0.08–0.64	0.005	0.34	0.12–0.97	0.044	Age, acquisition mode, methicillin resistant Sa, prosthetic infection, oral antibiotic switch, no optimal PAT, oxacillin, quinolone, time before surgery respected
	No	60	0.55	0.46–0.64	Ref						
Prolonged antibiotic combined therapy	Yes	39	0.42	0.33–0.53	0.44	0.26–0.72	0.001				
	No	25	0.64	0.49–0.79	Ref						
**Hospital organization**											
Hospitalization report signed by a senior physician	Yes	51	0.53	0.44–0.64	1.68	0.91–3.08	0.097				
	No	13	0.35	0.22–0.53	Ref						
**Outcomes of hospitalization**											
CIED implantation	Yes	8	0.69	0.42–0.92	1.72	0.82–3.61	0.153				
	No	56	0.46	0.38–0.56	Ref						
Oral antibiotic switch	Yes	9	0.26	0.14–0.44	0.31	0.15–0.62	0.001				
	No	55	0.57	0.47–0.67	Ref						
**Factors that could be associated with death**											
**Patient medical history**											
Prolonged bed rest	Yes	3	0.25	0.09–0.59	0.39	0.12–1.23	0.107				
	No	61	0.51	0.42–0.60	Ref						
Patient dependent for ADL	Yes	9	0.82	0.56–0.97	2.53	1.25–5.13	0.010				
	No	55	0.45	0.37–0.55	Ref						
Patient dependent for IADL	Yes	8	0.80	0.53–0.97	2.17	1.03–4.57	0.041				
	No	56	0.46	0.37–0.55	Ref						
Prior use of NSAIDs	Yes	8	0.80	0.53–0.97	2.81	1.33–5.92	0.007	3.60	1.59–8.15	0.002	Age, cerebral embolic complication, mode of acquisition, methicillin resistant Sa, prosthetic infection
	No	56	0.46	0.37–0.55	Ref						
**Severity upon admission**											
Quinolone resistance	Yes	8	0.80	0.53–0.97	2.00	0.95–4.20	0.067				
	No	56	0.46	0.37–0.55	Ref						
Use of vasoactive drug	Yes	24	0.65	0.50–0.80	2.36	1.42–3.92	0.001				
	No	40	0.42	0.33–0.52	Ref						
Use of dobutamine	Yes	12	0.75	0.53–0.92	2.61	1.38–4.91	0.003				
	No	52	0.45	0.36–0.54	Ref						
**Healthcare management**											
No probabilistic antibiotic therapy (PAT)	Yes	13	0.27	0.17–0.43	3.37	1.83–6.21	<0.001				
	No	51	0.61	0.50–0.71	Ref						
No efficient PAT	Yes	22	0.37	0.26–0.50	2.13	1.27–3.58	0.004				
	No	42	0.58	0.47–0.70	Ref						
No optimal PAT	Yes	53	0.45	0.37–0.55	2.23	1.16–4.29	0.016				
	No	11	0.73	0.50–0.92	Ref						
Not recommended antibiotic treatment	Yes	31	0.63	0.50–0.76	0.49	0.30–0.79	0.004				
	No	33	0.40	0.30–0.51	Ref						
Oxacillin	Yes	37	0.42	0.32–0.53	0.59	0.36–0.96	0.035				
	No	27	0.61	0.47–0.76	Ref						
Quinolone	Yes	27	0.40	0.30–0.53	0.62	0.38–1.01	0.057				
	No	37	0.56	0.45–0.69	Ref						
Non-performance of valve surgery when indicated	Yes	21	0.81	0.64–0.93	3.13	1.85–5.31	<0.001	1.85	1.01–3.39	0.046	Age, mitral location, methicillin resistant Sa, prosthetic infection, heart surgery and/or CIED extraction, time before surgery respected, palliative care
	No	43	0.40	0.32–0.50	Ref						
Time before surgery respected	Yes	13	0.31	0.19–0.47	0.45	0.25–0.83	0.011				
	No	51	0.56	0.47–0.67	Ref						
Extracardiac surgery indication	Yes	16	0.58	0.40–0.76	1.46	0.83–2.57	0.190				
	No	48	0.46	0.37–0.56	Ref						
Palliative care	Yes	22	1.00	1.00–1.00	6.67	3.79–11.73	<0.001				
	No	52	0.38	0.30–0.48	Ref						
**Hospital organization**											
Unavailable extra-cardiac surgery	Yes	4	0.80	0.42–0.99	2.39	0.87–6.58	0.093				
	No	60	0.47	0.39–0.56	Ref						
Unavailable valve surgery	Yes	9	0.75	0.50–0.94	2.08	1.03–4.22	0.042				
	No	55	0.46	0.37–0.55	Ref						
**Outcomes of hospitalization**											
Adverse event related to anticoagulant	Yes	5	0.83	0.48–0.99	2.40	0.96–5.99	0.061				
	No	59	0.47	0.38–0.56	Ref						
Surgical complication	Yes	8	0.80	0.53–0.97	2.15	1.02–4.52	0.044				
	No	56	0.46	0.37–0.55	Ref						
Nosocomial infection	Yes	17	0.68	0.50–0.85	1.85	1.06–3.22	0.031				
	No	47	0.44	0.35–0.53	Ref						

Quantitative variables: hazard ratio expressed as per unit increase of the variable. Quantitative variables: hazard ratio expressed as per 10 units-increase of the variable. IE, infective endocarditis; Sa, *Staphylococcus aureus*; GFR, Glomerular filtration rate; CIED, cardiac implantable electronic device; ADL, activity of the daily life; IADL, instrumental activity of the daily life; NSAIDs, non-steroidal anti-inflammatory drugs; PAT, probabilistic antibiotic treatment.

## Discussion

In this study, relying on a method derived from MMC, we identified three potential prognostic factors, two that were associated with higher mortality, i.e., the prior use of NSAIDs and the non-performance of valve surgery when indicated, and one that was associated with lower mortality, i.e., the decrease of vegetation size on antibiotic treatment.

To the best of our knowledge, no published study used a method derived from MMC to identify potential prognostic factors in SaIE. In most instances, it takes the form of a meeting of members of different medical teams who collectively try to retrospectively identify factors that may have contributed to a patient’s unfavorable outcome. The patient’s management is described and analyzed in an attempt to identify the causal factors of complications with the aim to improve the quality and safety of care, without judging individuals or looking for a culprit (21). In an attempt to standardize the course of MMC, Gregor and Taylor recommended that it be based on five essential components: (1) an adverse event (the case presented must include an adverse event that resulted from clinical decisions and/or care provided); (2) anonymity (individuals involved in the case must be afforded anonymity to allow for free and objective discussion); (3) critical analysis (based on reliable, objective data and careful attention to sources of bias in clinical decision making); (4) reframing understanding of errors to prevent their repetition; and (5) projection to practice change (19). Here, in an attempt to suggest practice changes to improve SaIE patients’ outcome, we organized a meeting of members of different medical backgrounds, i.e., infectious disease (MB, BH, AL, and BL), epidemiology (NA), and public health (NA), to collectively reframe the understanding of favorable or unfavorable prognosis relying on a critical analysis of all the data collected in the anonymized medical charts of some patients who died or survived from SaIE. And then, relying on a more classic quantitative approach, we raised hypotheses about factors that could be considered as prognostic factors.

We are aware that an association may not be causal. To establish epidemiologic evidence of a causal relationship between a presumed cause and an observed effect, Hill proposed to examine a set of nine criteria (22). We therefore examined our three potential prognostic factors versus these criteria (Table 5). NSAIDs were strongly associated with death, with a threefold higher risk of death in SaIE patients who had vs. those who had not received NSAIDs. To date, no study assessed the prognostic value of the use of NSAIDs in IE. However, NSAIDs are known to have multiple effects on innate and adaptive immunity (prostaglandin and leukotriene pathways) (23). Several studies showed that NSAID use could worsen the prognosis of infectious conditions, especially respiratory tract, osteoarticular and skin and soft tissue bacterial infection (24–26). The non-performance of valve surgery when indicated was associated with impaired prognosis in SaIE patients. This result was consistent with what was previously reported in other studies focusing on prognostic factors in IE and SaIE (1, 27, 28). In our study, as in previous studies (1, 27, 28), patients who did not undergo cardiac surgery despite indication were old, had many comorbidities, and presented with cardiac, infectious or embolic complications resulting in an unfavorable benefit-risk ratio for undergoing surgery. The decrease of vegetation size on antibiotic treatment was associated with a lower mortality. Decreasing vegetation size on antibiotic treatment was obtained by comparing diagnostic and last in-hospital echocardiography (which was censored by surgery in operated patient). The strength of this association was high, with a threefold decreased risk of death in SaIE patients in whom the vegetation size decreased. Regarding plausibility, our finding is consistent with prior results that showed a correlation between the size of the vegetation, the risk of embolism, and the risk of death in IE (13, 29–31). For this variable, as for the others, it seems essential to confirm our results in other studies. However, before that, it seems essential to find a standardized definition of a significant reduction in the size of the vegetation: how many millimeters? By what echocardiographic method? After what period of antibiotic treatment? An intervention to improve SaIE outcomes could be multidisciplinary meeting to adapt treatment when the vegetation size does not decrease during antibiotic treatment. For example, some authors believe that antibiotics other than those recommended should be tested in Sa infections, including IE, as they may have a better tolerability profile or even greater efficacy (32–38). However, these data are based on meta-analyses from biased observational studies and deserve to be confirmed in a large randomized controlled trial (39, 40). We have therefore identified three potential prognostic factors, two of which have never been identified before in endocarditis. In the future, it would seem interesting to confirm our results in an independent cohort. Ideally, a precise definition of each factor will have to be decided and the prognostic influence of each of them will be studied prospectively. Finally, if these results are confirmed, interventional projects could be developed to change the management of SaIE and improve its poor prognosis.

**TABLE 5:** The potential prognostic factors relevance in *Staphylococcus aureus* endocarditis assessed by the Hill criteria.

	Strength	Consistency	Specificity	Temporality	Biological gradient	Plausibility	Coherence	Experiment	Analogy
Prior use of NSAIDs	x			x		x			
Non-performance of valve surgery when indicated		x (1, 16, 27)		x		x (1, 16, 27)	x (1, 16, 27)		x
Decreasing of vegetation size on antibiotic treatment	x			x		x (13, 29–31)			

We acknowledge that our work may have some limitations. First, the patients’ medical charts may not include key prognostic information that was therefore missed by our qualitative approach. Second, due to the observational design, residual unmeasured confounding might still threaten the validity of our multivariate analyses. Third, due to the relatively low number of IE cases that were included in this study, we may not have been able to identify factors that had a genuine prognostic value. Fourth, the wording and definition of our candidate prognostic factors may be questioned and our results need now to undergo external validation in an independent dataset with standardized definitions to ensure reproducibility and therefore confirm the possible causality of the factors we identified.

## Conclusion

Our results suggest that the management of patients with SaIE could be improved by considering new prognostic factors. Although they need to be confirmed by additional studies, our findings indicate that the outcome of SaIE would be enhanced by limiting the use of NSAIDs, considering valve surgery when indicated even if the likelihood of postoperative death is high, and reconsidering the treatment strategy when the size of the vegetation does not decrease during the antibiotic treatment.

## Data availability statement

This raw data supporting the conclusions of this article are protected by the French law. Requests to access the datasets should be directed to the corresponding author.

## Ethics statement

This studies involving human participants were reviewed and approved by the French Commission Nationale de l’Informatique et des Libertés (CNIL-DR-2010-219). The patients/participants provided their written informed consent to participate in this study.

## Author contributions

BL, AL, MB, BH, and NA: conception and design of the study. BL, AL, MB, WN-S, BH, and NA: analysis and interpretation of data. All authors acquired of data, drafted the article or revised it critically for important intellectual content, and final approval of the version to be submitted.
