# Supplementary material for: Staphylococcus aureus endocarditis: Identifying prognostic factors using a method derived from morbidity and mortality conferences
Source: Front Med (Lausanne). 2022 Dec 6;9:1053278. doi: 10.3389/fmed.2022.1053278 (PMC9763316; doi:10.3389/fmed.2022.1053278)
Supplement: Supplementary file 1 [file Data_Sheet_1.pdf]

Supplementary Material

Infective endocarditis (IE) cases characteristics consisted of demographic, medical history, medications, IE mode of acquisition, clinical and initial biological data, and therapeutics used.

Demographic consisted of age and sex. Medical history included diabetes, hypertension, smoker, alcohol abuse, coronary heart disease, heart failure, valvular disease, peripheral arterial disease, chronic respiratory disease, gastric ulcer, chronic kidney disease, neurological disorders, malignant disease, hematological disease, liver diseases, immune deficiency, autoimmune disease, prosthetic valve, intracardiac device (pacemaker or implantable cardioverter-defibrillator), extracardiac prosthetic device and Charlson index.

Medications contained ongoing anticoagulant or antiplatelet agents.

Community acquired, healthcare related or intravenous drug use could be the mode of IE acquisition.

Clinical characteristics included initial Glasgow score, acute heart failure with cardiac surgery indication, cardiac conduction abnormalities, arterial aneurysm, vertebral osteomyelitis, arthritis, septic shock, extracardiac device infection and hemodialysis for acute kidney injury and embolic complications (cerebral or peripheral).

Initial biological characteristics involved white blood cells count in G/L, C-Reactive Protein in mg/L, glomerular filtration rate in mL/min/1.73m2, methicillin resistant Staphylococcus aureus (Sa).

Echocardiographic characteristics consisted of vegetation size, SaIE location, intracardiac abscess, new native valvular regurgitation/perforation, prosthetic endocarditis, and new prosthetic regurgitation/dehiscence.

Therapeutic characteristics included heart surgery, cardiac implantable electronic device removal, care or surgical complications (complications after cardiac surgery as prolonged ICU hospitalization ≥ 72 hours, acute kidney injury, second cardiac surgery needed, nosocomial infections and others….), extracardiac surgery.

Follow-up data consisted of survival status and date of death in case of occurrence.
